# Supplementary material for: miR-7 Controls the Dopaminergic/Oligodendroglial Fate through Wnt/β-catenin Signaling Regulation
Source: Cells. 2020 Mar 13;9(3):711. doi: 10.3390/cells9030711 (PMC7140713; doi:10.3390/cells9030711)
Supplement: Supplementary file 1 [file cells-09-00711-s001.pdf]

Supplementary Table 1: Raw data on *miR-7* knock-down, over-expression and rescue experiments in zebrafish.

Condition	N tot	% normal	% delayed / deformed	% dead
1) Not injected	90	95	0	5
2) mismMO (control)	38	94	0	6
3) mismMO+NCDP (control)	105	88	0	12
4) *mir7a*MO (mature form knock-down)	140	8	74	18
5) *lp7a*MO1/2/3 (immature form knock-down)	80	0	65	35
6) 7DP (*mir-7* duplex over-expression)	89	7	58	35
7) *mir7a*MO + 7DP (mature *miR-7* rescue)	117	81	9	10
8) *lp7a*MO1/2/3+7DP (immature *miR-7* rescue)	53	78	9	13

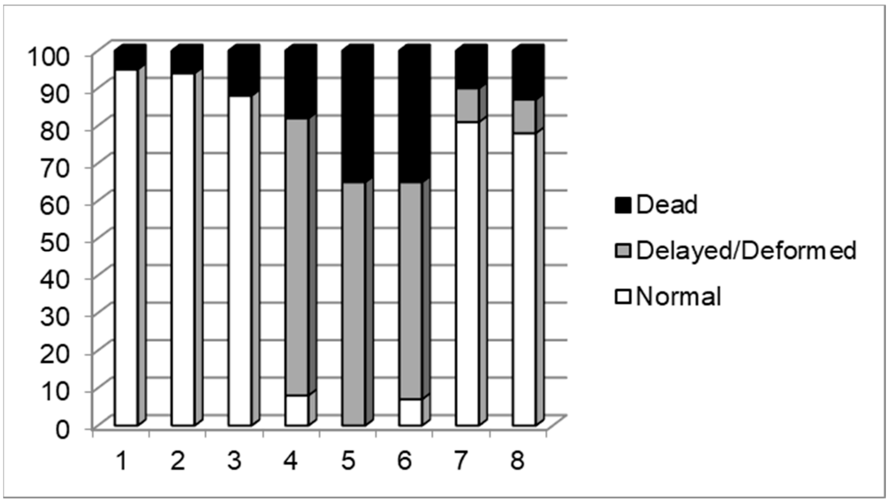

The table, with annexed chart, summarizes morphological phenotypes and mortality rates (at 2 dpf) of zebrafish embryos under *miR-7* knock-down, over-expression and rescue conditions.

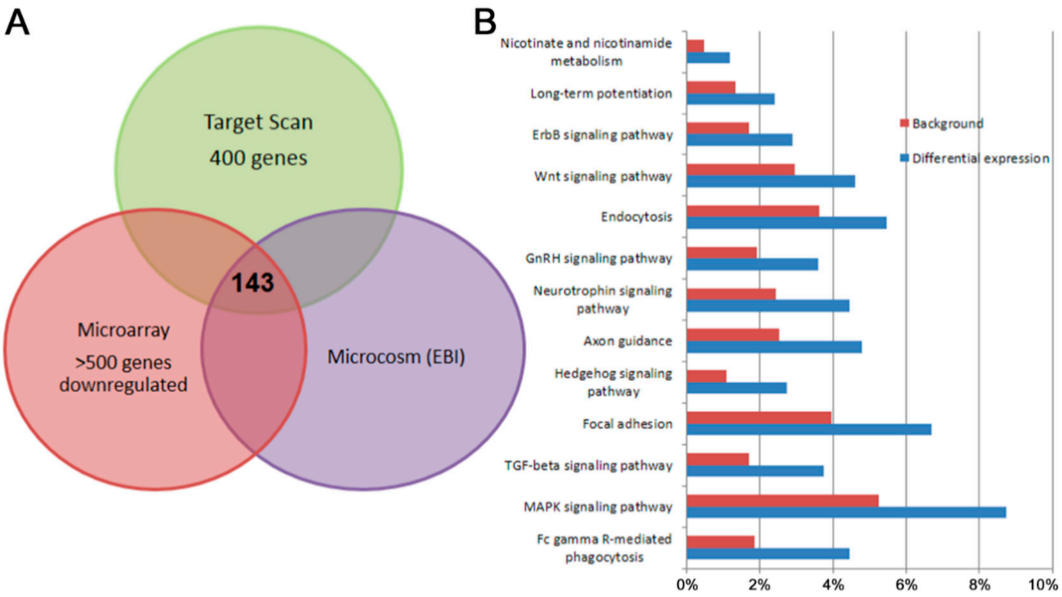

Figure S1. Profiling the downstream effectors of *miR-7*. (A) Human NPCs were transfected with 80 nM of *miR-7* duplex and control duplex in growth medium. RNA samples were collected 48 hrs post transfection and subjected to gene expression profiling using Ref-12 v4 Illumina bead chip. 143 potential targets were down-regulated by *miR-7* by microarray which also had PcT>0.1 in target scan. (B) Gene Ontology classification of genes down-regulated 2 days post transfection of *miR-7*. The fraction of differentially expressed genes (*mir7a* vs control duplex by 1.5 fold) belonging to each GO processes was compared to a fraction of the whole genome sorted into the same category.

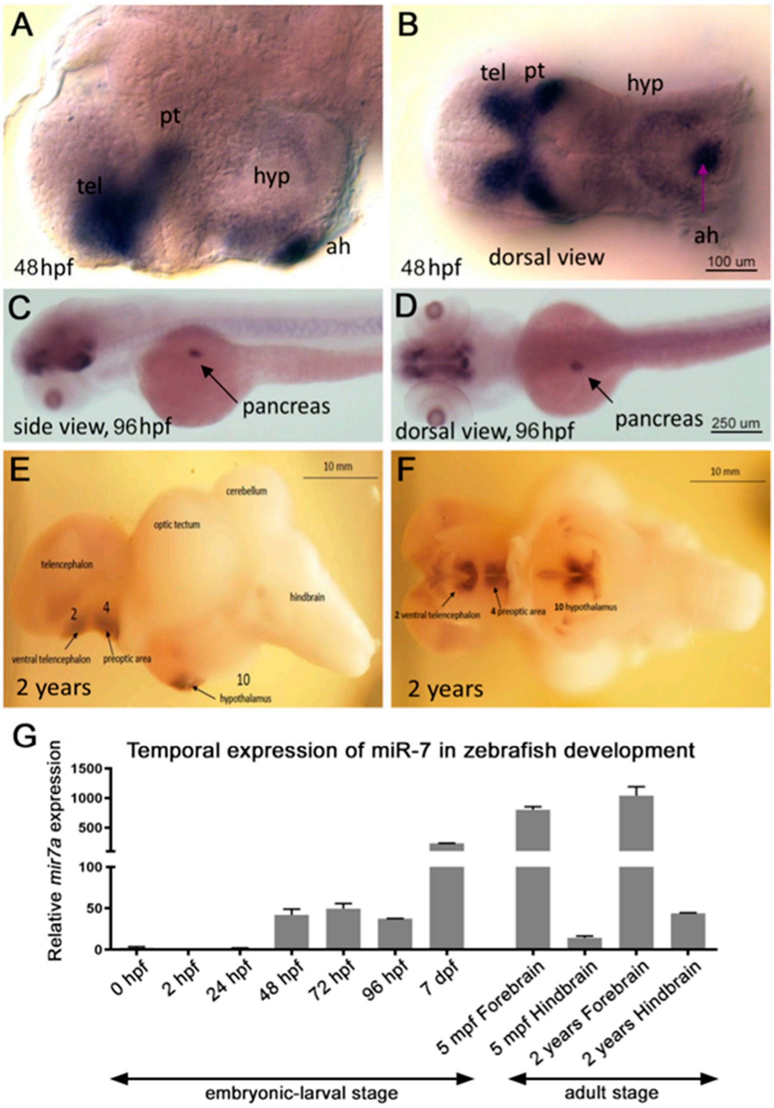

Figure S2. Spatiotemporal expression of *miR-7* during zebrafish embryogenesis. (A, B) Whole-mount LNA *in situ* hybridization of *miR-7* in zebrafish embryos at 48 hpf, in lateral (A) and dorsal view (B) of the head region, with anterior to the left. (C, D) Expression of *miR-7* at 96 hpf in the pancreas. Each image shows the expression pattern of *miR-7* in a representative embryo, out of 20 examined, in lateral (C) and dorsal (D) view, with anterior to the left; images mirrored for display reasons. (E, F) Expression of *miR-7* in a 2-year old zebrafish brain, with positive regions corresponding to those observed at embryonic stages. (tel) telencephalon, (pt) pituitary gland, (hyp) hypothalamus, (ah) adenohypophysis. (G) Quantification of *miR-7* transcription during zebrafish development: transcript levels were quantified by real-time PCR, normalized to internal controls (*18S* RNA), and presented as fold change ± SEM ($n \geq 3$) relative to the expression at 0 hrs.

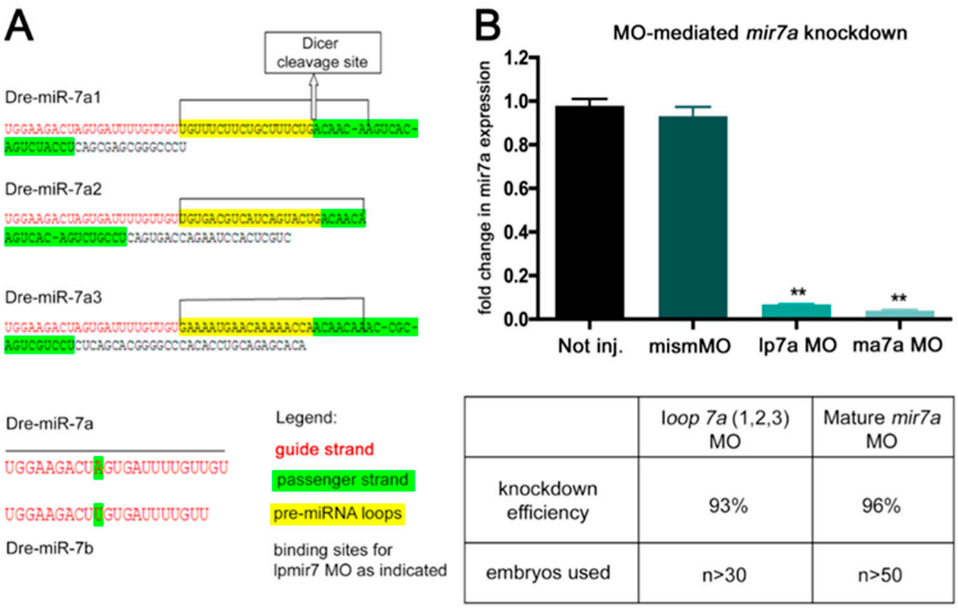

	loop 7a (1,2,3) MO	Mature mir7a MO
knockdown efficiency	93%	96%
embryos used	n>30	n>50

Figure S3. ***miR-7a* knockdown in zebrafish embryos causes a loss of *miR-7a* transcripts.** (A) Design of the morpholino (MO) antisense oligomers against the three loop precursor forms that block the Dicer cleavage site, and against the mature *miR-7a* sequence. (B) The graph indicates the qRT-PCR elucidating the effects of *miR-7a* morpholinos on the endogenous level of *miR-7a* at 48 hpf. Embryos were injected at 1-cell stage with control mismatch morpholino (misMO), with MOs against the loop forms (7a lpMO1/2/3), or against the mature *miR-7a* (7a mMO). Total RNA was obtained at 48 hpf. All expression values were normalized to 18S rRNA levels and presented as average fold change ± SEM ($n \geq 4$) relative to the expression values of not injected controls, where n is the number of biological replicates. The experiment was repeated four times with batch of 30 embryos in each experiment. Two tailed T-test results are indicated by (**) $P < 0.01$, relative to the not injected control.

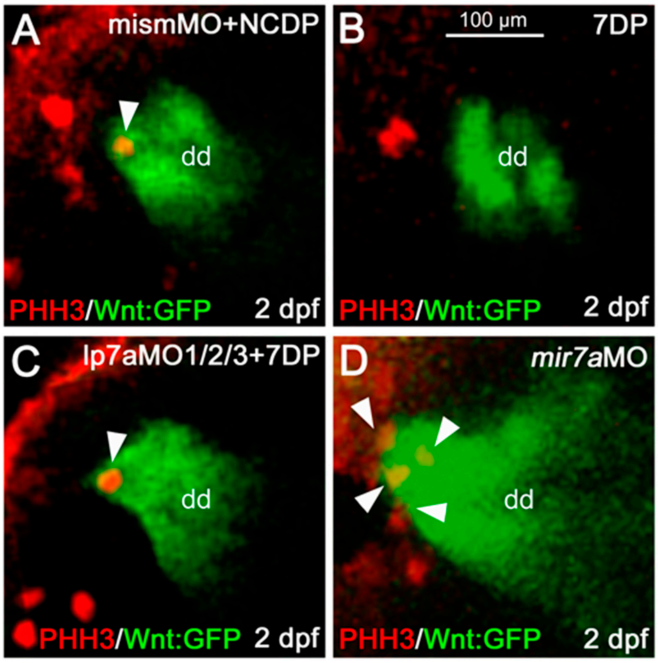

Figure S4. *miR-7a* regulates cell proliferation. (A) Cell proliferation analysis by anti-pHH3 staining (red signals) reveals on average few proliferating cells (1-2 cells in the displayed area, white arrowhead) within the diencephalic Wnt-responsive domain (green signals) in 2 dpf control embryos. (B) Decreased proliferation (no anti-PHH3 staining) is detected within the Wnt responsive domain of 7DP-injected embryos. (C) Co-injection of *mir7a*MO with the functional duplex (rescue condition) results in a control-like phenotype. (D) Increased proliferation (about 4-5 positive cells in the displayed area; white arrowheads) is detected within the Wnt-responsive domain of morphant embryos. All panels zoom on the dorsal diencephalic (dd) region of 2 dpf embryos in lateral view, anterior to the left. Displayed images represent the average phenotype from batches of n=20 embryos per condition; the experiment was performed in duplicate.

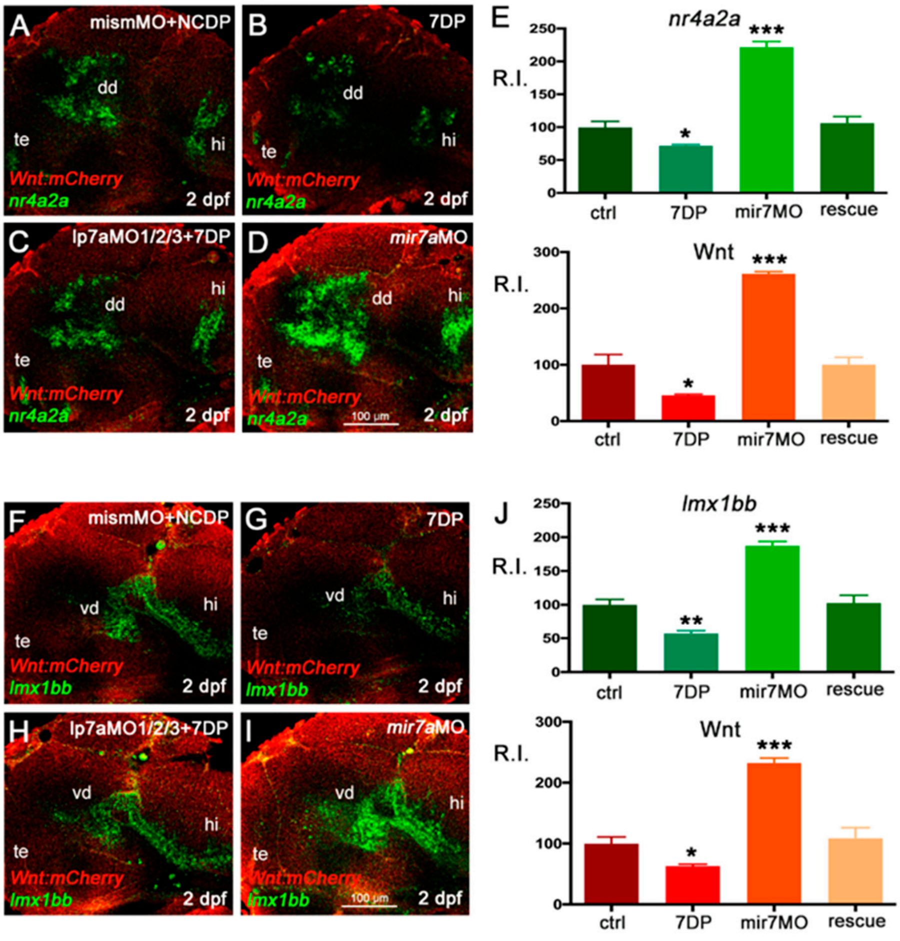

Figure S5. *miR-7a* regulates DA-associated neural precursor markers. (A-E) *miR-7* over-expression (7DP) and knockdown (*mir7a*MO) elicit decreased or increased expression, respectively, of the neural precursor marker *nr4a2a* (green), in parallel with decreased or increased Wnt-responsiveness (red); compare B and D with A (injected controls) and C (rescue). Signal quantifications are represented in E; (*) P<0.05; (***) P<0.001; n=5. (F-J*)* *miR-7* over-expression (7DP) and knockdown (*mir7a*MO) elicit decreased or increased expression, respectively, of the neural precursor marker *lmx1bb* (green), in parallel with decreased or increased Wnt-responsiveness (red); compare G and I with F (injected controls) and H (rescue). Signal quantifications are represented in J; (*) P<0.05; (**) P<0.01; (***) P<0.001; n=5. R.I.: relative intensity. te: telencephalon; dd: dorsal diencephalon; vd: ventral diencephalon; hi: hindbrain.

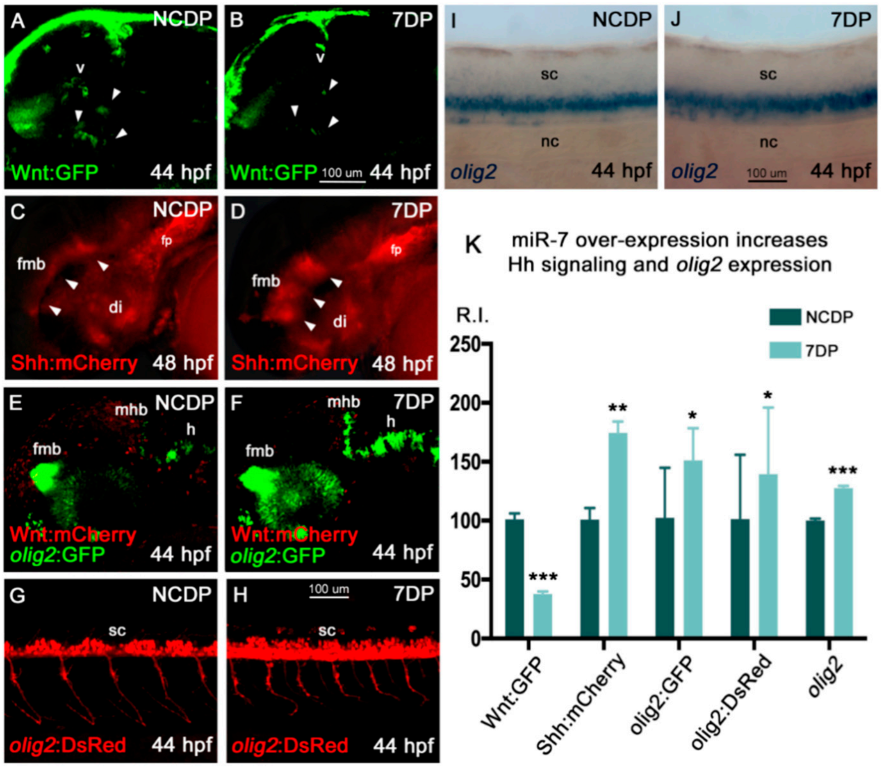

Figure S6: *miR-7* over-expression differentially regulates Wnt, Shh signaling and *olig2*. (A, B) Injection of the *miR-7* mimic (7DP) decreases expression of *Wnt:GFP* reporter in the ventral brain compared to NCDP-injected controls. (C, D) 7DP-injected embryos show an increase of *Shh:mCherry* reporter expression in the forebrain-midbrain boundary (fmb, white arrowheads) compared to NCDP injected controls. (E, F) 7DP injection in double transgenics *Wnt:mCherry/olig2:GFP* shows general decrease of Wnt-dependent mCherry nuclear signal and an increase of *olig2:GFP* transgene expression in the fmb, midbrain-hindbrain boundary (mhb) and hindbrain (h) region compared to NCDP controls. (G- J) 7DP injection induces an increase of *olig2:DsRed* transgenic protein (H) and endogenous *olig2* mRNA (J) expression in the ventral spinal cord of zebrafish embryos, compared to their control duplex (G,I). v: vessels; sc: spinal cord; nc: notochord; di: diencephalon; fp: floor plate. All panels are lateral views, anterior to the left. (K) Quantitative analysis of signal intensity in NCDP and 7DP embryos from all experimental series (n=5 measures per condition); (*) P<0.05; (**) P<0.01; (***) P<0.001. R.I.: relative intensity.

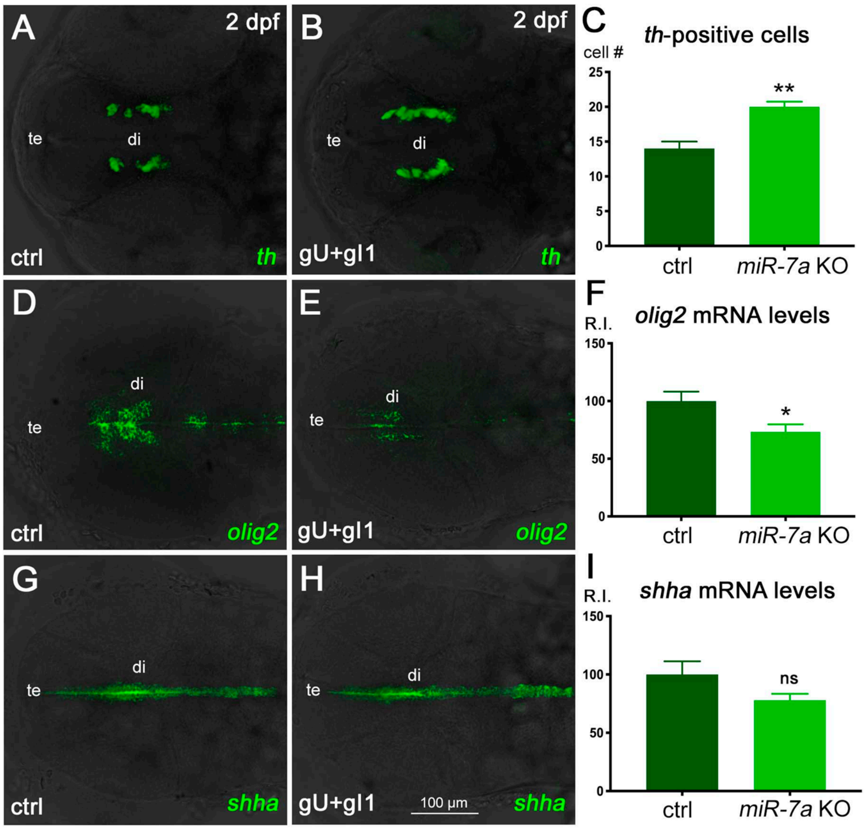

Figure S7: *miR-7* gene editing increases *th*-positive cells while decreasing *olig2* levels. (A-C) Crispants injected with *miR-7* specific guide RNAs (gU+gI1 combination) display an increased number of *th*-positive cells (B), compared to controls (A). Quantification in the C chart; (**), P<0.01; n=6. (D-F) *miR-7* crispants show reduced *olig2* expression (E), compared to controls (D). Quantification in the F chart; (*), P<0.05; n=6. (G-I) *miR-7* crispants (H) and controls (G) show comparable levels of *shha* expression. Quantification in the I chart; (n.s.), not significant; n=6. R.I.: relative intensity. te: telencephalon; di: diencephalon.

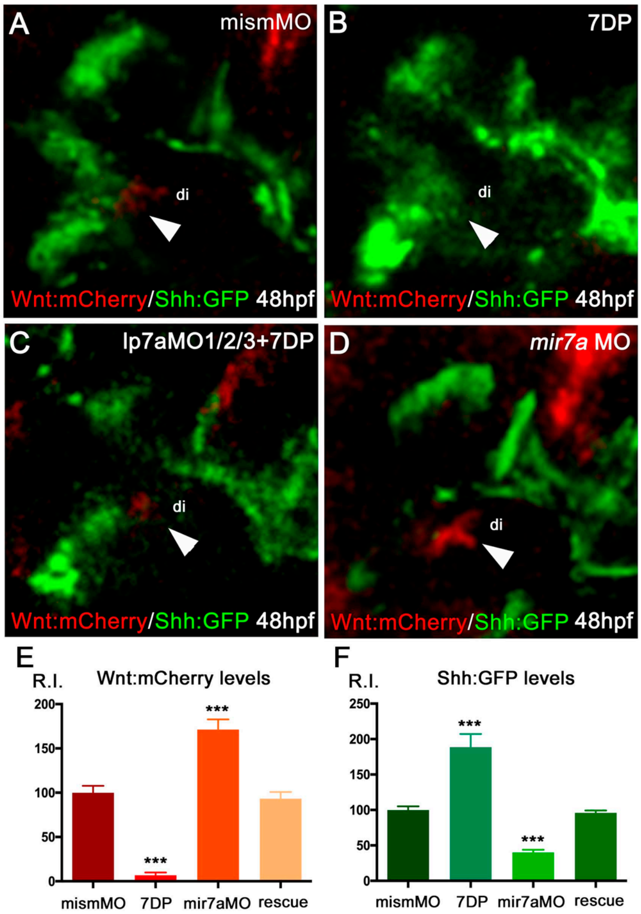

Figure S8: *miR-7* levels control the balance between Wnt- and Shh-responsive cells in the zebrafish brain. (A-D) *miR-7* over-expression (B) and knock-down (D) elicit opposite effects on Wnt- and Shh-responsive cells in the diencephalon (di) of Wnt/Shh double reporter zebrafish lines. The simultaneous knock-down and over-expression (C) rescues both signals to levels comparable with the control (A). The white arrowhead indicates the ventral posterior tuberculum/hypothalamic region of 48 hpf embryos in lateral view, anterior to the left. Signal quantifications are represented in E and F; (***) P<0.001; n=5. R.I.: relative intensity.

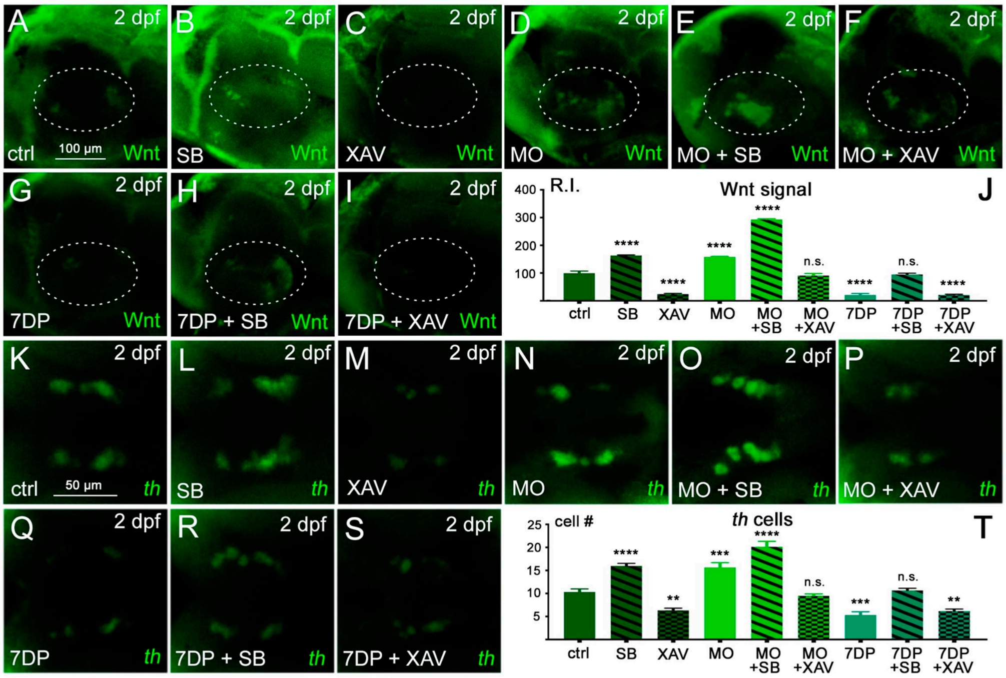

Figure S9: Simultaneous manipulation of *miR-7* and Wnt levels regulates the number of diencephalic *th*-positive cells. (A-T) XAV939 (XAV)-mediated inhibition or SB216763 (SB)-induced over-activation of Wnt signaling, in combination with *miR-7* downregulation (MO) and overexpression (7DP), can increase, decrease or rescue Wnt-responsiveness (A-I, quantification in J) and the physiological number of *th*-positive cells (K-S, quantification in T) in the diencephalic region (encircled in A-I and zoomed in K-S) of 2 dpf zebrafish brains. (A-I) lateral and (K-S) dorsal views, anterior to the left. (****) P<0.0001; (***) P<0.001; (**) P<0.01; n.s. not significant; n=6 per condition. R.I.: relative intensity.

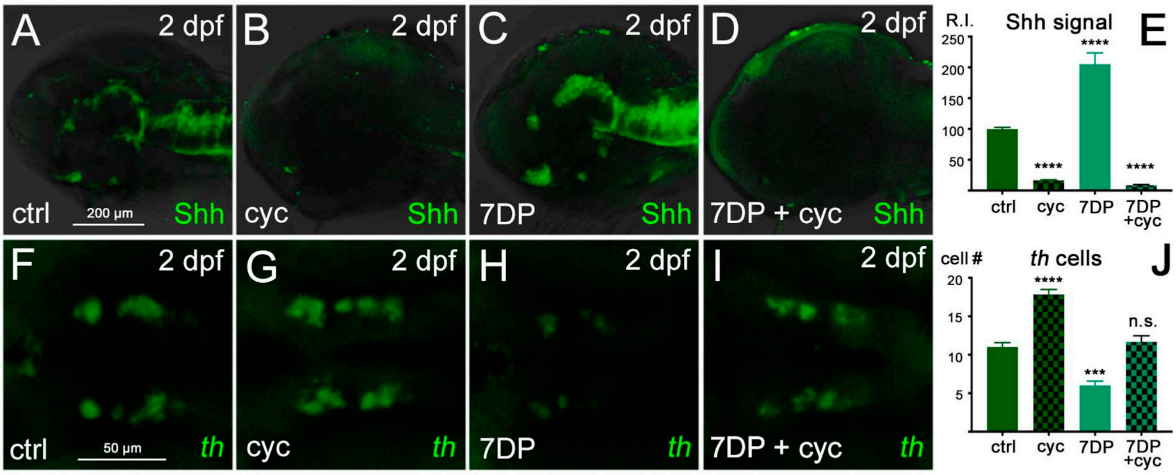

Figure S10: Simultaneous manipulation of *miR-7* and Shh levels regulates the number of diencephalic *th*-positive cells. (A-J) *miR-7* over-expression (7DP) can rescue the physiological number of diencephalic *th*-positive cells (compare I with F) under cyclopamine (cyc)-mediated Shh inhibition (compare B and D with A). A-D are lateral views and F-I dorsal views of 2 dpf zebrafish brains, anterior to the left. Quantifications in E and J. (****) P<0.0001; (***) P<0.001; n.s. not significant; n=6 per condition. R.I.: relative intensity.
